# Changes in Medical Education during the COVID-19 Pandemic

**DOI:** 10.31138/mjr.31.4.376

**Published:** 2020-12-28

**Authors:** Ilke Coskun Benlidayi

**Affiliations:** Cukurova University Faculty of Medicine, Department of Physical Medicine and Rehabilitation, Adana, Turkey

**Keywords:** COVID-19, medical education, telemedicine

The coronavirus disease 2019 (COVID-19) pandemic has disrupted routines of daily living in several ways. It changed the way we socialise, work, and communicate. The pandemic has also disrupted teaching practices at medical schools and hospitals. The cessation of in-person classes, cancellation of clinical clerkships, and non-urgent elective surgeries, over-occupation of training hospitals during the pandemic contributed to the disruption of medical education.^1^

**Figure:**
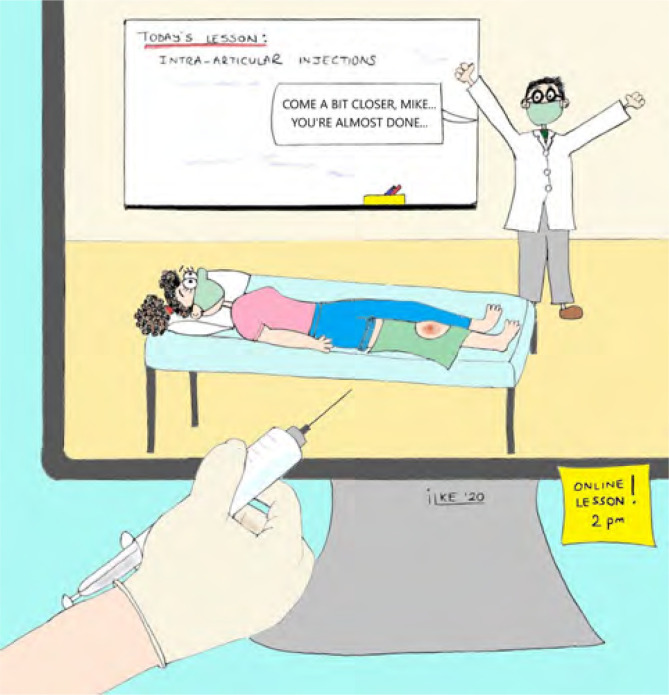


Beginning from the early days of the pandemic, educators and academicians worldwide have tried to explore different ways for communication and teaching. Platforms that would enable delivery of synchronous lectures and asynchronous recordings have been rapidly adopted by medical universities.^2^ Online platforms emerged as the most appropriate way for disseminating knowledge. Teaching meetings via teleconferencing might comprise lectures, interactive sessions, and students’ topic/case presentations.^3^ Further arrangements are required for senior medical students to promote their clinical skills and knowledge.^4^ In this regard, teleconferencing may also help to demonstrate surgical and other medical procedures.^5^ Online teaching/learning has both pros and cons. It is more flexible in terms of location and time, more engaging and time-efficient. On the other hand, there are some disadvantages such as technical issues and lack of proper patient-student interaction.^2^

Direct in-person care is unquestionably the most beneficial option in medical education.^2^ Medical institutions have been trying to discover ways to resume patient-student communication during the pandemic. Chandra et al. reported a virtual clinical experience for senior medical students in an Emergency Medicine clerkship.^6^ The supervised virtual call-backs for patients recently examined in the Emergency department were performed by the students. This virtual clinical program has received favourable feedback from involved students and patients.^6^ A survey by Escalon et al. revealed that 86.5% of physiatrists in the United States were practicing via telemedicine, and 92.5% of Physical Medicine and Rehabilitation residents were participating in virtual learning during the COVID-19 pandemic.^7^ Engaging students in telemedicine approaches varying from phone triage to electronic visits, follow-up visits, and consultations can help to bridge the gap in medical education and patient-student interaction.^8^

Medical education via teleconferencing and virtual workshops cannot substitute for hands-on clinical training. However, this brand-new way of learning and teaching might serve as an acceptable option in such a time of pandemic. So, let us give Mike some courage to sort his hard task out!
